# 

**Published:** 1859-01

**Authors:** 


### INDEX.

Ague epidemic at Cannington, Mr. Haviland on, 266
Alcohol, its place and power, 32
Army, hygiene of the, 1, 132; clothing of, 90; mortality in, 139
Aspley Guise, climate of, 312
Bird on Erysipelas, 253
Bombay, sanitary condition of, 373
Cattle, prize, diseases of, 137
Central or local action, 134
Chemical manufactories, effect on health, 250
Child-murder, Dr. Ryan on, 165
Cholera, Dr. Pinkerton on spread of, among pilgrims at Juggernauth, 248
Cigar-making, 325
Climate, effect on consumption, 137
Clothing of women, 341; of soldiers, 90
Coffins, air-tight, 133
Constantinople, sanitary state of, 270
Consumption, hygienic treatment of, 93
Consumptive patients, special exercises for, 247
Cowhouses in London, 91
Death-rates of districts, 129
Death-rate of England, 209
Egypt, a residence for invalids, 338
Epidemic and endemic diseases, local reports of, 94, 187, 297, 391
Epidemics and their everyday causes, Mr. Cox on, 60, 254
Epilepsy, hygienic treatment of, 43
Falcony's process, 252
Fever, continued, type of, 249; diarrhœa from foul water, Mr. Cox on, 371
Flax-dressing, 322
Food grains of India, 334
Fur-dyeing, 322
Health of England in 1857, 86; 1856, 328
Hemp-dressing, 321
Hospital hygiene, 113
Hygiene, Dr. Pickford's, 141
Idiotcy, causes of, 140, 225
India, food grains of, 334
Infant hygiene, 339
Injurious occupations, 317
Kitchens, underground, Dr. F. J. Brown on injurious effects of, 58
Lemon-juice, care in administering, 338
Life, prolongation of, in 18th century, 135
Life and labour, 317
Mortality in army, 139; among occupations, 46
Moustache and beard in the navy, Dr. Duigan on, 361
Naval hygiene, 338
Occupations, mortality in, 46
Physiology, place of, in general education, 23
Post Office, Dr. W. Lewis on health of officers and men in, 49
Printers, consumption among, 248
Prisoners at Wakefield, Mr. Milner on alterations in weight of, 348
Rags, Mr. J. Murray on working among, 363

Rational medicine, 44
Reports on cities, towns, and districts, 270, 373
Ridge on health and disease, 252
Sand-paper, manufacture of, 319
Sanitary legislation, Mr. Rumsey's views on, 41; notes of, 111, 207, 316, 410
Sanitary science, past and present, 243
Sewer emanations, Dr. Barker on, 70; Dr. Letheby on, 275
Sewerage of towns, 131
Sinope, Mr. Radcliffe on topography, meteorology, and sanitary condition of, 145
Skin, prevention of diseases of, 45
Social science transactions, 129
Swine pestilence, 234
Teeth, decayed, effects of retention, 252
Thames, effect of, on health, 47; purification of, 142, 386
Transmission of disease between men and animals, 246
Ventilation of St. James's Hall, 144; self-acting, Mr. Radcliffe on, 343
Walking-stick making, 320
Water of London, drinking, 138
Water, Dr. J. B. Harrison on contamination with lead, 185; foul, Mr. Cox on fever and diarrhœa from, 371

RICHARDS, 37, GREAT QUEEN STREET.

### NOTES AND CORRESPONDENCE.

THE Editor is anxious to give free scope to the expression of opinion, but will not hold himself responsible for the opinions of those of his contributors who attach their names to the papers.

In concluding the Fourth Volume of the SANITARY REVIEW, the Editor cannot thank too warmly the many earnest friends who have supported him by their labours. Through their assistance this Review has now found its way into all parts of the world where literature exerts its power; and the cause of sanitary science, helped in its progression at home, has, we had almost said, been originated in some of the colonial possessions of England. In acknowledging the assistance rendered him in his labours, the Editor must once more ask for a continuance of the same ready and willing aid.

Papers intended to be read at the Epidemiological Society should be forwarded to Dr. McWilliam, C.B., Trinity Square, Tower Hill. We have only to mention that last session there was rather a deficiency of papers, to ensure from our Epidemiological contributors all the needful. Amongst the many learned societies of the metropolis, the Epidemiological is the last that should be forgotten. Its objects are so extended, and its usefulness so obvious, that we hope to see the day when a representative member of it shall be found in every city and town in the kingdom. The terms of membership are one guinea per annum for members resident in London, and one guinea for life for non-resident members.

VENTILATION OF CLOTHES. A perforated gusset for insertion into men's garments, to allow the escape of gaseous matter, has been sent to us for examination by Messrs. Silver and Company. This gusset, introduced into clothes, is very useful and comfortable. We are informed that it is under consideration to introduce this invention in the manufacture of clothes for the army, and for other bodies of men actively employed in the public service. The idea is very simple, but it would be of unquestionable service especially to men obliged to make long and fatiguing marches in hot climates.

We had prepared a report on Mr. Falcony's method of preserving the dead, for this number, but are prevented from inserting the report by want of space. Meantime, we can speak of the plan as being very successful and useful.

### BOOKS RECEIVED.

Agricultural Review: a Journal of Rural Economy and Industrial Science. Published monthly; Vol. I., No. 9. Dublin: T. H. Saunders. November, 1858.

Annual Medical Report of the Kent Lunatic Asylum at Barning Heath, Maidstone, for the Year 1857-58, ending July 4. pp. 31. Maidstone: 1858.

Armstrong (Alexander, M.D., R.N.) Observations on Naval Hygiene and Scurvy, more particularly as the latter appeared during a Polar Voyage; pp. 117. London: Chnrchill, 1858.

Den Hygieiniske Congres i Kjöbenhavn, Juli 1858. Kjöbenhavn: 1858.

Dickinson (Joseph, M.A., M.D., F.R.S.) Egypt and Nubia: their Climate, Character, and Merits, as a Winter Residence for Invalids. pp. 34. Liverpool: H. Greenwood, 1858.

Dobell (Horace, M. D.) Demonstrations of Diseases in the Chest, and their Physical Diagnosis. pp. 115. London: Churchill, 1858.

Evans (Conway, M.D. Lond.) Reports on the Sanitary Condition of the Strand District, London. Illustrated with Forty Tables and Diagrams. London: Churchill, 1858.

Guy (W. A., M.B. Cantab.) On the Production and Identification of Crystals of Arsenious Acid, and Crusts of Metallic Arsenic. [From Dr. Beale's *Archives of Medicine*, No. III., 1858.] pp. 13. London, 1858.

Harrison (R. E.) What is Congelation? or the Benumbing Influence of Cold in producing Insensibility to Pain in Dental Operations popularly explained. pp. 90. London: Churchill, 1858.

Hornemann (Dr. E.) Hygieiniske Meddelelser og Betragtninger. Kjöbenhavn: 1856-7-8. (Four Numbers.)

Ladies' National Association for the Diffusion of Sanitary Knowledge. 1. How to manage a Baby. 2. How to feed a Baby with the Bottle. 3. The Evils of Wet-nursing: a Warning to Mothers. 4. The Evils of Perambulators: a Word to Mothers. 5. The Cheap Doctor: a Word about Fresh Air. 6. Why do not Women swim? a Voice from Many Waters. Pamphlets. London: Groombridge and Sons, 1858.

MURCHISON (C., M.D.) Remarks on the Classification and Nomenclature of Continued Fevers. [Reprinted from the *Edinburgh Medical Journal*, October 1858.] Edinburgh: 1858.

M. M. Experience of Factory Life. Reprinted from *The Waverley*, a Working-woman's Journal. pp. 48. London: 1858.

NORTHCOTE (A. B., F.C.S.) On the Constitution of Thermophyllite. From the *Philosophical Magazine* for October 1858.

NORTHCOTE (A. B., F.C.S.) On the Brine-Springs of Cheshire. From the *Philosophical Magazine* for December 1857.

NUNN (Thomas W., F.R.C.S.) Observations and Notes on the Arteries of the Limbs. pp. 27. London: Churchill, 1858.

ROOKE (T. M., M.D.Lond.) Marine Sanatoria: their Objects and Requirements, with Special Reference to the Northern Sea-bathing Infirmary at Scarborough. pp. 14. Scarborough: S. W. Theakstone, 1858.

Sanitary Aspect of Bombay.

WARD (F. O.) Purification of the Thames. A Letter addressed to William Coningham, Esq., M.P. [Printed by Mr. Coningham for Circulation.] Second Edition. pp. 14. London: Renshaw, 1858.

WATSON (J. Forbes, A.M., M.D., F.C.S.) Remarks on the Development of the Resources of India. [Introductory chapter from work on the Food Grains of India.] pp. 36. London: Smith, Elder & Co., 1858

### IN EXCHANGE.

Literary Gazette.
Psychological Journal.
British Medical Journal.
Ranking and Radcliffe's Abstract of the Medical Sciences.
Chemist.
Veterinarian.
Asylum Journal of Mental Science.
Quarterly Journal of the Chemical Society.
Quarterly Journal of Dental Science.
Scottish Review.
Edinburgh Medical Journal.
Edinburgh Veterinary Review.
Glasgow Medical Journal.
Dublin Hospital Gazette.
American Medical Monthly.
American Journal of the Medical Sciences.
New York Journal of Medicine.
Montreal Medical Chronicle.
Charleston Medical Journal.
Gazette Médicale de Paris.
Journal de Physiologie.
Aerzliches Intelligenz-Blatt.
Medical and Surgical Reporter.
Journal of Practical Medicine and Surgery.
Gazette Médicale de l'Orient.
Hygieiniske Meddelelser og Betragtninger.

*Is now Published, price 16s.*

### THE ASTLEY COOPER PRIZE ESSAY,

ON THE

### CAUSE OF THE

### COAGULATION OF THE BLOOD:

WITH A PRACTICAL APPENDIX.

BY

BENJAMIN W. RICHARDSON, M.D., L.R.C.P.,

PHYSICIAN TO THE ROYAL INFIRMARY FOR DISEASES OF THE CHEST.

### Contents:

CHAPTER I. Historical Memoranda on the supposed Causes of Coagulation of the Blood.

CHAPTER II.—Cause of the Phenomenon of Coagulation: present position of the Question.

CHAPTER III.—The Author's Personal Observations and Deductions on the Condition of the Blood in the Body under certain Physiological and Pathological States.

CHAPTER IV.—Experimental Inquiry into the Physical Agencies influencing the Coagulation of the Blood.

CHAPTER V.—Experimental Inquiry into the Chemical Agencies influencing the Coagulation of the Blood.

CHAPTER VI.—Experimental Inquiry into the Chemical Agencies influencing the Coagulation of the Blood (continued).

CHAPTER VII.—Phenomenon of the Buffy Coat.

CHAPTER VIII.—Summary of Conclusions and Propositions.

APPENDIX.

1.—Superalkaline Conditions of the Blood in Relation to Disease.
2.—Ammonia as an Exhalation from the Body.
3.—Experiments, shewing the Effect of Lactic Acid on Animal Bodies (with Coloured Plates, shewing the stages of Endocarditis).
4.—Deposition of Fibrin in the Heart and Blood-vessels during Life.
5.—The Alkalies and Acids as Remedial Agents.
6.—Application of Ammonia to the Experiment of Transfusion of Blood.
7.—On some Conditions of the Blood after Death, in relation to Medico-Legal Inquiries.
8.—List of Authors.

***The Essay is Illustrated with numerous Wood Engravings, and with Coloured Plates by Tuffen West.***

"We shall merely give an outline of what we may justly call Dr. Richardson's great discovery, feeling certain that all who desire to keep themselves up to the professional science of the day will study the work for themselves....It is with extreme pride and satisfaction that we congratulate our countryman upon the appearance of his great work."—*Medical Times and Gazette.*

"We recommend to our readers a careful perusal of the whole treatise, which we look upon as not less remarkable for its high value as a model of logical research and a study for experimental physiologists, than it is memorable in the annals of our scientific literature for its luminous truths and satisfactory results."—*The Lancet.*

"The essay of Dr. Richardson affords an instructive lesson, as well as encouragement, to all who are engaged in investigating the phenomena of nature."—*British Medical Journal.*

"Nous proclamons sans hésiter que ce livre est destiné à prendre place parmi les œuvres scientifiques les plus remarquables de notre époque."—*Journal de la Physiologie.*

London: JOHN CHURCHILL, New Burlington Street.

